# A case series of OMI: time for revisiting STEMI/NSTEMI ECG criteria

**DOI:** 10.1186/s43044-025-00688-2

**Published:** 2025-09-30

**Authors:** Edwin Adhi Darmawan Batubara, Bambang Widyantoro, Siska Suridanda Danny, Dafsah Arifa Juzar

**Affiliations:** Department of Cardiology and Vascular Medicine, Faculty of Medicine, Universitas Indonesia, National Cardiovascular Center Harapan Kita, Jakarta, Jakarta, Indonesia

**Keywords:** STEMI/NSTEMI, OMI/NOMI, Delayed reperfusion

## Abstract

**Background:**

The current STEMI criteria fail to detect roughly one‐third of occlusive MI (OMI). STEMI criteria demonstrated only a sensitivity of around 21% for OMI. Compared to STEMI, patients with NSTEMI-OMI have higher short-term and long-term all-cause mortality and longer reperfusion delays. This case series presents three NSTEMI patients with OMI and a delayed reperfusion strategy.

**Case presentation:**

We present three cases of patients initially diagnosed with NSTEMI who were later found to have occlusive myocardial infarction (OMI) based on angiographic findings, all of whom underwent delayed reperfusion strategies. The first two cases shared similar clinical profiles, presenting with typical infarct angina, elevated cardiac enzymes, and regional wall motion abnormalities on echocardiography. Their electrocardiograms showed bifascicular blocks, right bundle branch block (RBBB) with left posterior fascicular block (LPFB) in the first case, and RBBB with left anterior fascicular block (LAFB) in the second. Both were classified as high-risk NSTEMI and scheduled for an early invasive approach. Angiography revealed total occlusions in the OM1 and left main arteries, respectively. In the third case, the patient presented with new-onset angina and elevated cardiac biomarkers, but without ECG features fulfilling STEMI or high-risk OMI criteria. However, due to persistent chest pain despite initial treatment in the emergency department, an immediate invasive strategy was pursued. Coronary angiography revealed a total occlusion in the proximal left anterior descending (LAD) artery.

**Conclusions:**

These three cases underscore the diagnostic challenge of identifying occlusive myocardial infarction (OMI) in patients presenting with acute coronary syndrome (ACS) when relying exclusively on traditional STEMI criteria. They emphasize the need to recognize alternative ECG markers indicative of acute coronary occlusion, as failure to do so may result in delayed reperfusion and subsequently worse clinical outcomes compared to patients who receive timely intervention based on prompt STEMI recognition.

**Supplementary Information:**

The online version contains supplementary material available at 10.1186/s43044-025-00688-2.

## Background

Myocardial infarction (MI) terminology has developed vastly over the last three decades. The electrocardiogram (ECG) Q wave/non-Q-wave terminology was first introduced before thrombolytic therapy, which revealed that thrombotic therapy without revascularization cannot reverse ongoing myocardial necrosis and led to large territory infarction [1–3]. The fibrinolytic therapy trialists meta-analysis revealed thrombolysis therapy was related to a lower mortality rate only in patients with ST elevation (STE) or bundle branch block (BBB) and treated up to 12 h from onset. However, the definition criteria for these patients were poorly described. The STEMI/Non-STEMI terminology was formally formed in the 2000 ACC/AHA guidelines [3, 4]. The STEMI terminology has been used to identify total occlusive MI (OMI). This resulted in reduced reperfusion delays and lower mortality rates. A study by Neto et al. showed the pooled sensitivity and specificity for STE from ECG to be 43.6% and 96.5%, respectively, for detecting total occlusion [5]. However, challenges were faced with patients who did not match the STEMI criteria but had OMI. This terminology is still, by definition, a “Non-STEMI,” according to the guidelines, which will lead to a delay in reperfusion and poorer outcomes [1]. Approximately, around 25–30% of NSTEMI patients have OMI, which is associated with a higher risk of major cardiovascular events. A study from Kola et al. exhibited that NSTEMI-OMI patients had significant catheterization delays and angiographic findings, PCI rates, and complications similar to STEMI [2]. This case study aims to present three NSTEMI patients with OMI and a delayed reperfusion strategy.

## Case presentation

### Case I

**Fig. 1:**
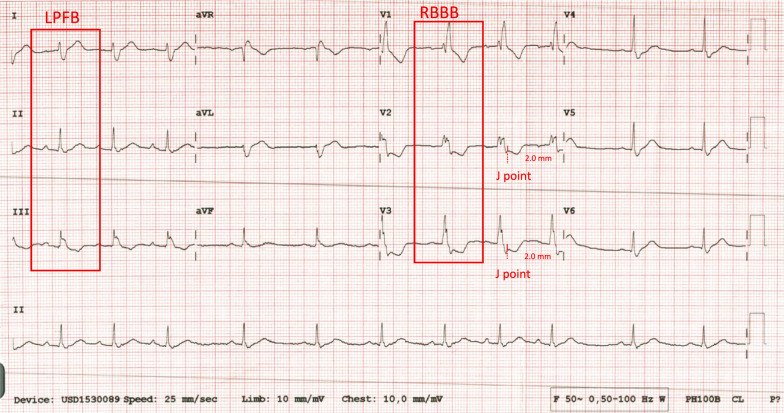
ECG (4 h onset) demonstrated sinus rhythm with right bundle branch block with ST depression in V2–V3 and left posterior fascicular block

**Fig. 2:**
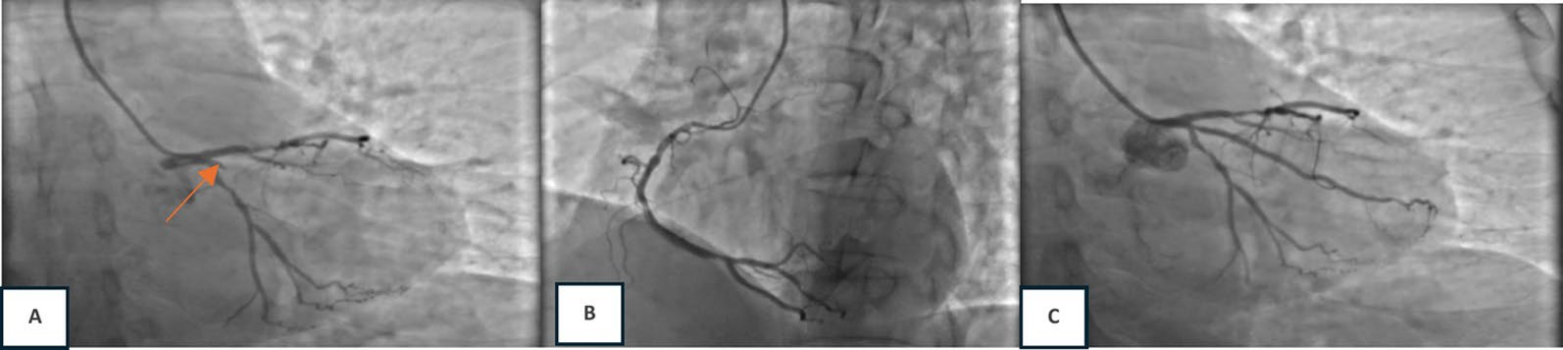
Early PCI procedure. **a** A diagnostic angiography revealed diffuse stenosis 70–80% at proximal LAD, diffuse stenosis 80–90% at proximal LCx, and total occlusion at proximal OM1 with TIMI Flow 0 (red arrow). **b** Right coronary artery (RCA) was dominant with discrete stenosis of 30–40% at osteal. **c** Successful PCI with implantation of 2.25 × 22 mm coronary stent at proximal OM1 with TIMI Flow 3

A 51-year-old male came to the emergency department (ED) with a history of chest pain four hours before admission. The pain was moderate and worsening, described as heavy and tightness, with a scale of 5/10, radiating to the neck and jaw. He also experienced nausea and cold sweats. The patient had a history of hypertension, dyslipidemia, a familial history, and smoking. On physical examination, the patient presented with a normal mental status, blood pressure of 129/60 mmHg, a heart rate of 60 bpm, oxygen saturation of 99% on room air, and a respiratory rate of 17 breaths per minute. Physical examination revealed normal findings. ECG examination showed sinus rhythm, 100 bpm, right axis deviation, right bundle branch block with ST depression in V2–V3, and left posterior fascicular block (Fig. 1). A chest X-ray was performed, revealing a CTR of 56% with no signs of congestion or infiltrate. Laboratory examination revealed a creatinine level of 0.96 (eGFR 96) with an increased high-sensitivity troponin T (Hs Trop T) level of 33 ng/L and a serial two-hour level of 110 ng/L. Bedside echocardiography in the ED found an ejection fraction (EF) of 58%, TAPSE of 17 mm, and a hypokinetic anterior–anterolateral mid to basal segment with good cardiac valves. The patient was diagnosed with a high risk of NSTEMI. The Global Registry of Acute Coronary Events (GRACE) score was 138, and the CRUSADE bleeding score was 9, with dyslipidemia and hypertension. The patient was treated with lovenox (60 mg twice daily SC), clopidogrel (75 mg once daily), miniaspi 80 mg (once daily), atorvastatin 40 mg (once daily), captopril (12.5 mg thrice daily), and isosorbide dinitrate (3 × 5 mg thrice daily). The patient was then admitted to the ICVCU ward and scheduled to undergo early percutaneous coronary intervention (PCI). The following day (24 h after admission), angiography showed total occlusion in obtuse marginal 1 with significant stenosis in the proximal left circumflex artery (LCx) and proximal left anterior descending (LAD) artery (Fig. 2). Stenting was performed on obtuse marginal 1 (OM1), and the result was good with TIMI Flow 3. The patient was discharged on day 4 to the general ward in good clinical condition with stable hemodynamics.

### Case II

**Fig. 3:**
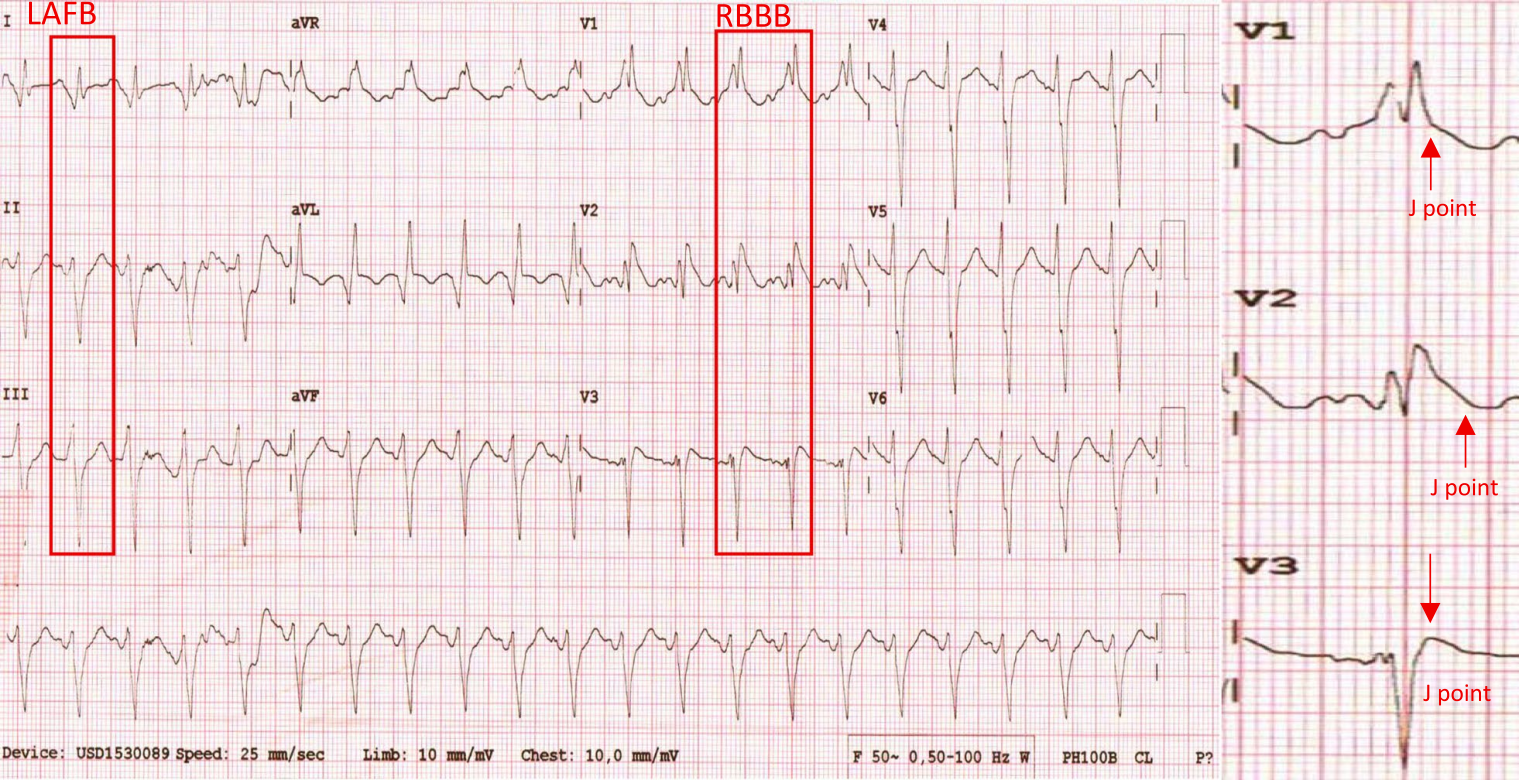
ECG (24 h onset) demonstrated sinus tachycardia with right bundle branch block and left anterior fascicular block

**Fig. 4:**
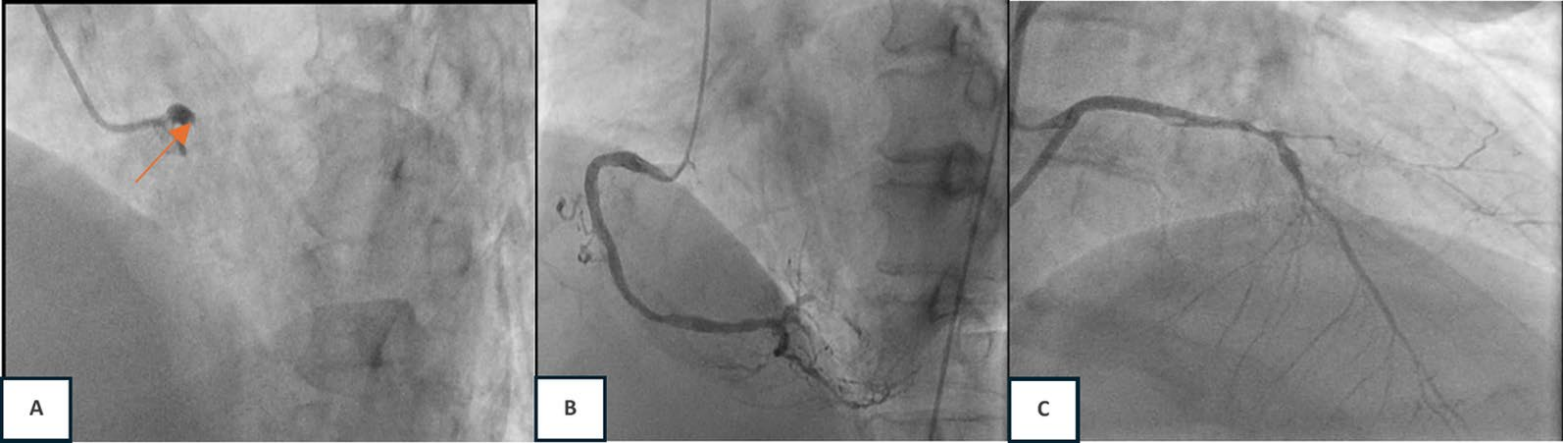
Early PCI procedure. **a** A diagnostic angiography revealed total occlusion at the proximal left main with TIMI flow 0 (red arrow). **b** RCA was dominant with subtotal occlusion at distal PL. **c** A successful PCI with POBA and thrombectomy was performed, and the result was TIMI Flow 3, residual stenosis, and thrombus

A 59-year-old man with a history of heavy smoking, diabetes mellitus, and hypertension presented to the ED with substernal chest pain 24 h ago. The chest pain was described as squeezing and tightness. The pain was severe and worsening, with a scale of 7/10, radiating to the left hand and back. The pain was not relieved by resting. The patient also reported breathlessness, nausea, and diaphoresis. The patient had a history of coronary artery disease in the last 1 year. On physical examination, the patient appeared to have a normal mental status, with blood pressure of 130/85 mmHg, a heart rate of 125 bpm, an oxygen saturation of 98% in room air, and a respiratory rate of 28 breaths per minute. A neck examination revealed elevated jugular venous pressure (JVP). A lung examination showed bilateral rales. Initial ECG showed sinus tachycardia, 125 bpm, left axis deviation, right bundle branch block, and left anterior fascicular block (Fig. 3). A chest X-ray revealed a CTR of 68%, an elongated aortic segment, and signs of congestion. Laboratory examination revealed increased HS Trop T (6519 ng/L) with increased creatinine 2.32 (eGFR 33) and blood glucose 386. Bedside echocardiography in ER found EF 25%, TAPSE 14 mm, akinetic basal anterior and anteroseptal, mid anteroseptal, anterior, anterolateral, dan inferolateral, apico anterior, and lateral. Other segments were hypokinetic, AR moderate, MR mild, and TR mild. The patient was then diagnosed with high risk of NSTEMI (GRACE score 130 and CRUSADE score 53), acute decompensated heart failure due to coronary artery disease, DM type II with hyperglycemia, hypertension, and renal insufficiency. The patient was treated with lovenox (60 mg twice daily SC), ticagrelor (90 mg twice daily), miniaspi (80 mg once daily), atorvastatin 40 mg (once daily), captopril (6,25 mg thrice daily), isosorbide dinitrate (3 × 5 mg thrice daily), furosemide (40 mg twice daily intravenous), and intravenous insulin drip and planned to perform early PCI. The patient was sent to the cath laboratory the next day. The angiography (24 h after admission) showed total occlusion in the left main and subtotal stenosis in distal PL. Early PCI with balloon angioplasty (POBA) and thrombectomy with aspiration catheter were performed on the left main coronary artery; then, the result was good with TIMI flow 3 with residual stenosis and residual thrombus (Fig. 4). After the procedure, the patient was transferred to the CVCU and discharged uneventfully 9 days later to the general ward.

### Case III

**Fig. 5:**
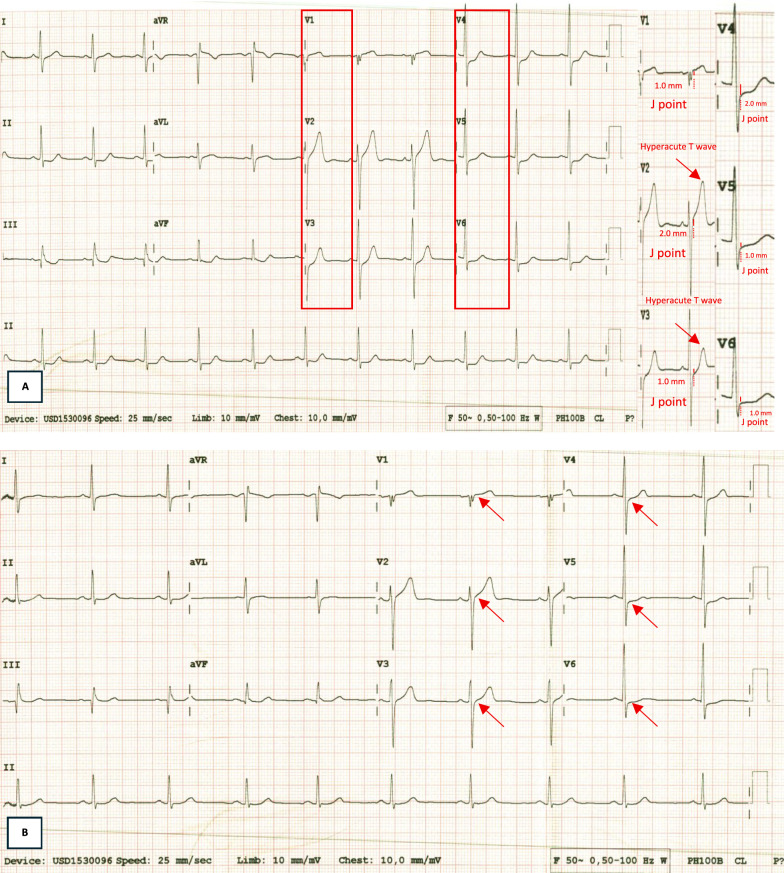
ECG **a** (5 h onset) sinus rhythm, 69 bpm, Normo axis, P wave normal, PR interval of 160, QRS duration of 80 ms, Q wave (-), ST depression in V3–V6, subtle ST elevation in V1–V2, and prominent T wave in V2–V3. **b** A serial ECG (6 h onset) examination revealed normalization of ST depression in V3–V6 with a prominent T wave in V2–V3

A 41-year-old male presented at our hospital with chest pain five hours before admission. The pain was severe, described as squeezing or aching, with a scale of 7/10, and radiating to the left hand. The pain wasn't relieved by resting. The patient had a history of hypertension and was an active smoker. On physical examination, the patient appeared compos mentis, with blood pressure of 143/96 mmHg, heart rate of 72 bpm, oxygen saturation of 99% room air, and respiratory rate of 22 per minute. Physical examination was within normal limits. ECG examination showed sinus rhythm, 69 bpm, ST depression in V3–V6, subtle ST elevation in V1–V2, and prominent T wave in V2–V3. (Fig. 5a) Serial ECG examination revealed normalization of ST depression in V3–V6 with a prominent T wave in V2–V3. (Fig. 5b). A chest X-ray was performed, revealing a cardiothoracic ratio of 53%, without signs of congestion or infiltrate. Laboratory examination revealed creatinine 1.01 (eGFR 96) with increased HS Trop T (242 ng/L). Bedside echocardiography in the ER revealed an EF of 60%, TAPSE of 22 mm, and a global normokinetic pattern with good cardiac valves. The patient was diagnosed with high risk of NSTEMI (GRACE score 128 and CRUSADE score 9) and hypertension. The patient was treated with lovenox (60 mg twice daily SC), ticagrelor (90 mg twice daily), miniaspi (80 mg once daily), atorvastatin (40 mg once daily), captopril (6,25 mg thrice daily), amlodipine (10 mg once daily), and isosorbide dinitrate (3 × 5 mg thrice daily).

**Fig. 6:**
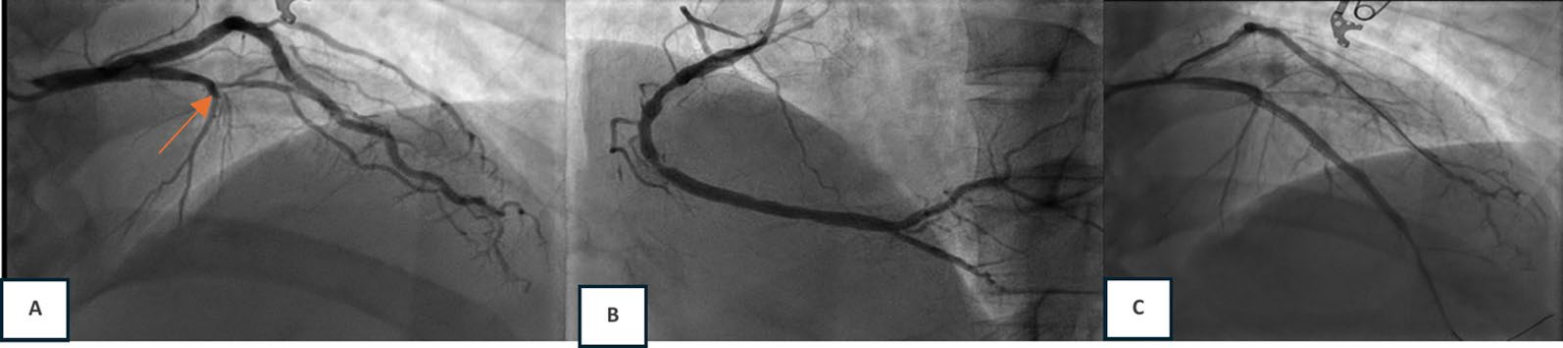
Immediate invasive strategy procedure. **a** A diagnostic angiography revealed total occlusion at the proximal LAD with TIMI flow 0 (red arrow). **b** RCA was dominant, with diffuse stenosis at 70% in the mid. **c** Successful PCI with implantation of 3.5 × 38 mm coronary stent and 2.5 × 32 mm coronary stent was performed with the result TIMI flow 3

After being treated with sublingual nitrate in the ED, the patient felt persistent chest pain, and the patient was sent to the cath laboratory for an immediate invasive strategy. The angiography (6 h after admission) showed total occlusion in the proximal LAD and significant stenosis in mid-RCA. Stenting was performed on proximal LAD, and the result was good with TIMI flow 3 (Fig. 6). The patient was discharged on day 2 to the general ward in good clinical condition with stable hemodynamics.

## Discussions

We have presented three case series to the emergency department with NSTEMI, manifesting with OMI, and a delayed reperfusion strategy. Smith and Meyers (2018) described occluded MI using OMI terminology. According to the OMI paradigm, MI is categorized by all available information based on whether the patient has acute coronary occlusion or near occlusion without collateral circulation, resulting in imminent transmural infarction without reperfusion [6]. When only retrospective data is available, Meyers et al. also suggested that the OMI be defined as (1) a reduced flow acute culprit lesion, (2) an acute culprit lesion accompanied by a normal TIMI flow grade 3 and a high peak troponin. and (3) a high peak troponin and a new regional wall motion abnormality for patients who do not have an angiography [1, 6]. In 2013, the American College of Cardiology Foundation/American Heart Association (ACCF/AHA) published guidelines for ECG criteria in patients with STEMI [7]. The European Society of Cardiology (ESC) 2017 STEMI guidelines stated ST-segment elevation (measured at the J-point), which suggests ongoing coronary artery acute occlusion. However, it only applies if the ST-segment is elevated in two contiguous leads. [8] Our three patients were classified as NSTEMI with OMI, and none of our ECG patients met ST elevation criteria. However, the angiography result showed total occlusion in the culprit lesion. When comparing STEMI and NSTEMI/OMI, Meyers discovered that the STEMI criteria overlooked 40% of NSTEMI/OMI cases and that NSTEMI/OMI patients had peak troponins similar to those of STEMI, indicating they were at risk for avoidable delayed reperfusion [1]. The DIFOCCULT study also showed 28.2% of NSTEMI patients re-classified as having OMI [9]. According to current criteria, the first case’s ECG showed a right bundle branch block and a left posterior fascicular block. There was insufficient evidence to classify it as an ST elevation MI. However, the catheterization demonstrated OM1 blockage. The second case ECG revealed a right bundle branch block with a left anterior fascicular block. The diagnosis of type 1 myocardial infarction was confirmed by catheterization, which also showed a left main artery blockage. There are factors to consider linked to ischemia and infarction when an RBBB is present. It should be possible to predict a complete coronary artery blockage with a close examination of the ECG. A study by Petr et al. showed that left main artery occlusion was manifested in 9 of 35 patients whose ECGs showed RBBB with or without LAFB. Fascicular block (RBBB + LAFB or LPFB) may occur without the usual STE because of large territorial infarcts (caused by proximal LAD coronary artery occlusion or left main coronary artery occlusion). Mortality from STEMI is also higher in new RBBB compared to new LBBB, and it is even higher in fascicular block [8, 10].

According to the 2022 ACC Expert Consensus, a few individuals with acute coronary blockage would go unnoticed if only the STEMI ECG criteria were applied to a 12-lead ECG. Hyperacute T waves or ST-segment elevation < 1 mm, if paired with reciprocal ST-segment depression, may indicate coronary vessel occlusion. The ECG should be closely examined for subtle changes that may be the first signs of vessel occlusion [11]. In 2025, Ricci et al. also published a review of ECG patterns of OMI. The following should be taken into consideration: modified Sgarbossa criteria (patients with paced rhythm or LBBB; Aslanger Pattern); depressions of ST in V1–V3 indicated posterior MI; acute pathological Q waves (Q waves associated with minimal ST elevation, not related to a previous myocardial infarction); any inferior ST elevation with any ST depression or T wave inversion in aVL; terminal QRS distortion and new-onset bifasicular block [2, 12]. Our first and second cases were administered with typical angina infarct elevated troponin, and the ECGs also revealed bifascicular block, which was also included in the ECG patterns of OMI.

The third case showed that the patient's ECG displayed a precordial swirl sign pattern, revealing subtle ST elevation or hyperacute T waves in V1–V2, with reciprocal ST depression in V5 and/or V6, suggestive of an OMI [13]. Catheterization demonstrated an left anterior descending (LAD) occlusion. When combined with the patient’s persistent presentation at the time of the chest pain, risk factors, past medical history, elevated troponin levels, and worsening chest pain following medical treatment, these findings showed that the patient was deemed to be in very high-risk stratification and needed immediate catheterization. In this case, the hyperacute T wave inversion in this ECG is not typical for ischemia, as it is characterized by a T-tall morphology, with symmetry, broad-based, non-tented, and non-pointed features. Additionally, the onset of chest pain did not correlate with the evolution of ECG ST elevation findings. A comprehensive approach should be used when assessing patients to detect OMI [2, 14, 15]. It has recently been demonstrated that an artificial neural network can diagnose OMI with twice the sensitivity of the STEMI criteria while maintaining the same specificity. An artificial intelligence (AI) design for OMI detection demonstrated superior performance sensitivity and specificity when compared to STEMI criteria, with an area under the curve of 0.938 [95% CI: 0.924–0.951], according to a study by Herman et al. [16].

Our first and second cases, classified as high-risk NSTEMI, underwent early invasive angiography within 24 h. The third case was of very high risk, with a time to immediate invasive angiography of less than 6 h. Herman et al. study showed patients with NSTEMI-OMI have higher short-term and long-term all-cause mortality and longer reperfusion delays than those with STEMI. Approximately, 9.9% of STEMI patients and 18.1% of NSTEMI-OMI patients experienced all-cause death in one year [17]. These results support the necessity for quicker and more precise NSTEMI-OMI identification. Rapid reperfusion can reduce myocardial damage and improve clinical results.

A Nune’s study developed a proposed algorithm for treating chest discomfort in the emergency room. The important parameters include STE criteria, other ECG abnormalities, quick ECG interpretation, and first clinical assessment. The methodology also takes into account the potential for false-negative OMI cases, highlighting the significance of keeping an eye out for dynamic changes in the ECG or refractory chest pain as signs that prompt reperfusion therapy is necessary [11]. Therefore, if applied to our patients, OMI can be detected in our first and second cases due to typical infarct angina with elevated enzymes, and ECGs showed bifasicular block. In our third case, the ECG demonstrated a precordial swirl pattern, which can benefit from immediate reperfusion in cases. It is essential to consider that implementing the new OMI categorization will likely encounter difficulties. The number of patients with OMI whose presenting ECG shows no ST elevation is rising. Currently, available AI models or expert ECG training could have identified these cases, and the patients would have gained an advantage from rapid reperfusion therapy [18].

## Conclusions

An estimated 25–30% of patients initially diagnosed with NSTEMI actually have underlying occlusive myocardial infarction (OMI), which is often only recognized during delayed cardiac catheterization. These patients carry a higher risk of major adverse cardiovascular events compared to those with STEMI. Prompt identification of OMI is therefore critical, particularly in ACS patients who do not fulfill traditional STEMI criteria. Early recognition of specific ECG patterns indicative of OMI is key to identifying this high-risk subgroup. A comprehensive diagnostic approach, including detailed clinical assessment, careful ECG interpretation, biomarker evaluation, and echocardiographic imaging, should be meticulously applied to facilitate timely reperfusion and improve outcomes.

## Supplementary Information

Additional file1 (JPEG 168 KB)

Additional file2 (JPEG 163 KB)

Additional file3 (JPEG 169 KB)

## Data Availability

No datasets were generated or analyzed during the current study.

## Abbreviations

AI: Artificial intelligence
ACC: American College of Cardiology
AHA: American Heart Association
ECG: Electrocardiogram
ED: Emergency department
EF: Ejection fraction
ESC: European Society of Cardiology
GRACE: The global registry of acute coronary events
HS: Trop T high-sensitivity troponin T
LAD: Left anterior descending artery
LAFB: Left anterior fascicular block
LBBB: Left bundle branch block
LCx: Left circumflex artery
LPFB: Left posterior fascicular block
MI: Myocardial infarction
NOMI: Non occlusive myocardial infarction
NSTEMI: Non-ST elevation myocardial infarction
OMI: Occlusive myocardial infarction
PCI: Percutaneous coronary intervention
RCA: Right coronary artery
RBBB: Right bundle branch block
STEMI: ST elevation myocardial infarction
TIMI: Thrombolysis in myocardial infarction
