# Supplementary figures and images for: A case series of OMI: time for revisiting STEMI/NSTEMI ECG criteria
Source: Egypt Heart J. 2025 Sep 30;77:90. doi: 10.1186/s43044-025-00688-2 (PMC12484458; doi:10.1186/s43044-025-00688-2)

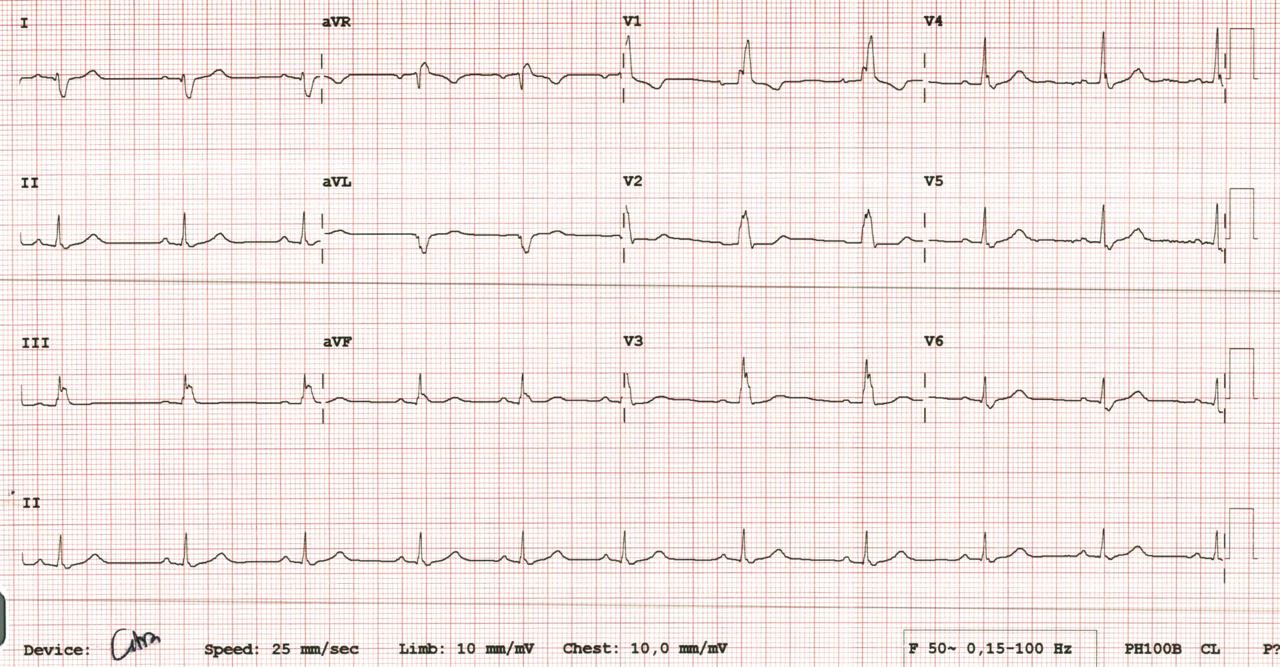

Supplement: Supplementary file 1 — Additional file1 (JPEG 168 KB) [file 43044_2025_688_MOESM1_ESM.jpeg]

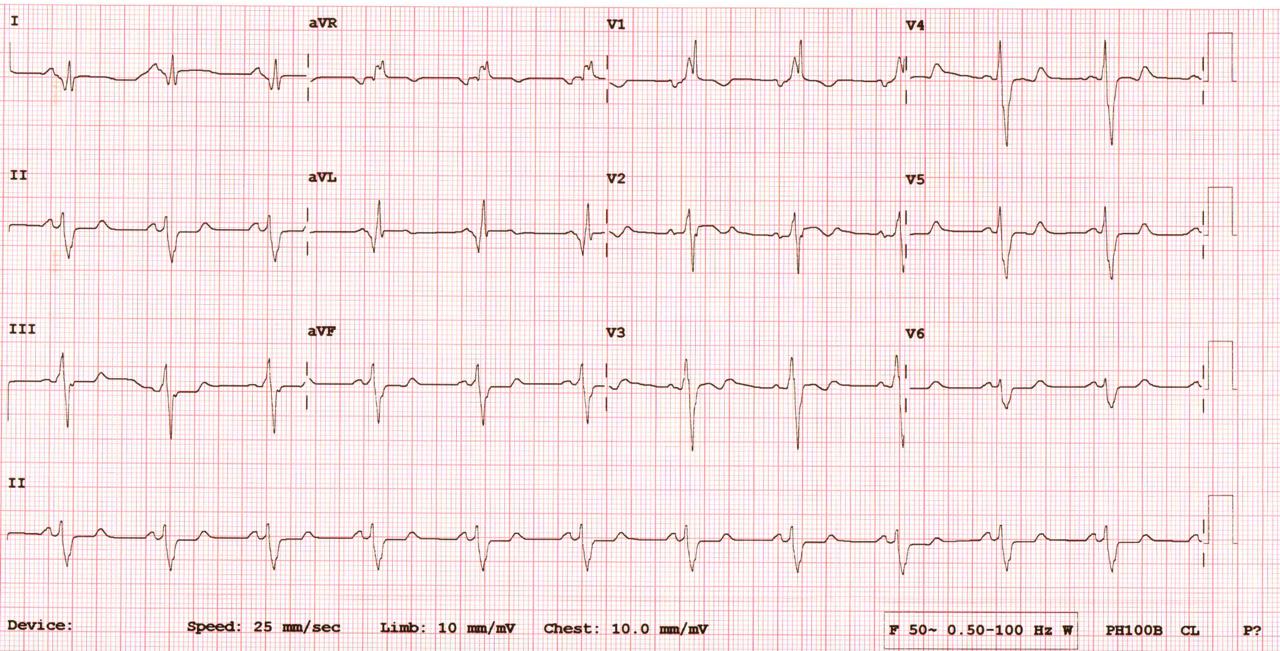

Supplement: Supplementary file 2 — Additional file2 (JPEG 163 KB) [file 43044_2025_688_MOESM2_ESM.jpeg]

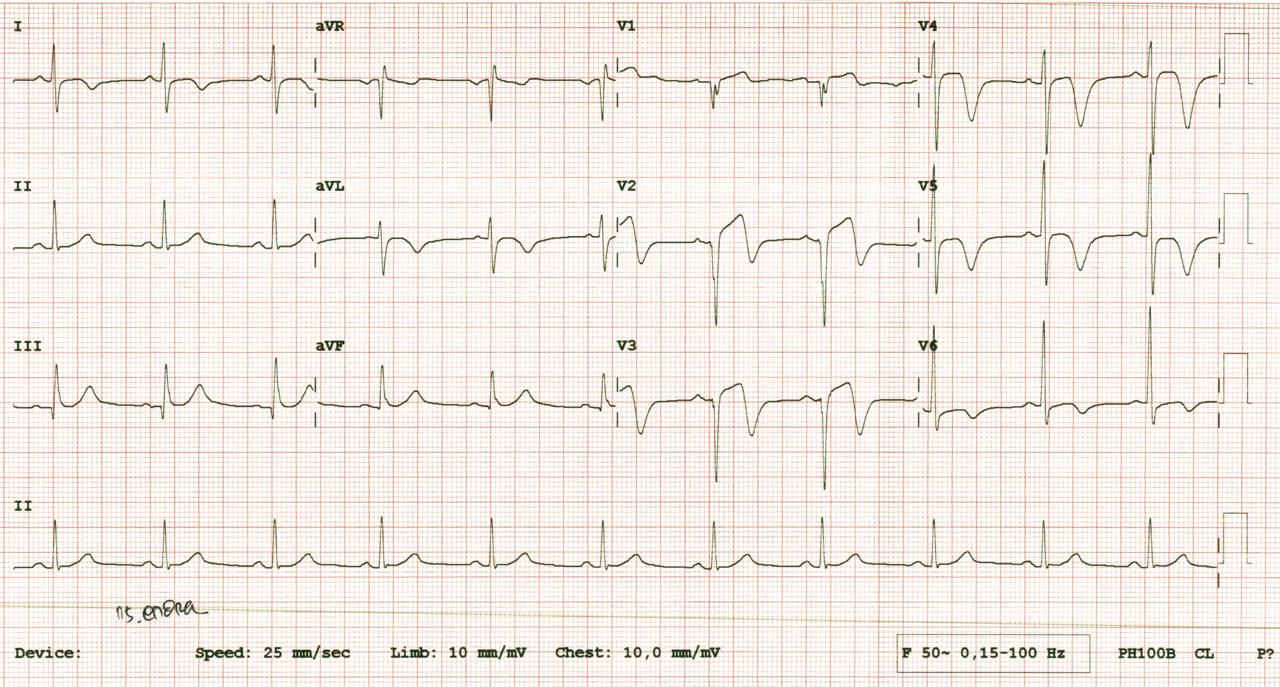

Supplement: Supplementary file 3 — Additional file3 (JPEG 169 KB) [file 43044_2025_688_MOESM3_ESM.jpeg]
